# Xylella fastidiosa: climate suitability of European continent

**DOI:** 10.1038/s41598-019-45365-y

**Published:** 2019-06-20

**Authors:** Martin Godefroid, Astrid Cruaud, Jean-Claude Streito, Jean-Yves Rasplus, Jean-Pierre Rossi

**Affiliations:** CBGP, INRA, CIRAD, IRD, Montpellier SupAgro, Montpellier, France

**Keywords:** Ecological epidemiology, Ecological modelling

## Abstract

The bacterium *Xylella fastidiosa (Xf)* is a plant endophyte native to the Americas that causes diseases in many crops of economic importance (grapevine, *Citrus*, Olive trees etc). *Xf* has been recently detected in several regions outside of its native range including Europe where little is known about its potential geographical expansion. We collected data documenting the native and invaded ranges of the *Xf* subspecies *fastidiosa*, *pauca* and multiplex and fitted bioclimatic species distribution models (SDMs) to assess the potential climate suitability of European continent for those pathogens. According to model predictions, the currently reported distribution of *Xf* in Europe is small compared to the large extent of climatically suitable areas. The regions at high risk encompass the Mediterranean coastal areas of Spain, Greece, Italy and France, the Atlantic coastal areas of France, Portugal and Spain as well as the southwestern regions of Spain and lowlands in southern Italy. The extent of predicted climatically suitable conditions for the different subspecies are contrasted. The subspecies *multiplex*, and to a certain extent the subspecies *fastidiosa*, represent a threat to most of Europe while the climatically suitable areas for the subspecies *pauca* are mostly limited to the Mediterranean basin. These results provide crucial information for the design of a spatially informed European-scale integrated management strategy, including early detection surveys in plants and insect vectors and quarantine measures.

## Introduction

The bacterium *Xylella fastidiosa (Xf)* is a plant endophyte native to the Americas, which develops in up to 300 plant species including ornamental and agricultural plants^1^. *Xf* is transmitted between plants by xylem-feeding insects belonging to several families of Hemiptera (Aphrophoridae, Cercopidae, Cicadellidae, Cicadidae and Clastopteridae)^2^. *Xf* causes severe plant pathologies leading to huge economic losses^3^ *e.g*., the Pierce’s disease of grapevines PD^4^, the olive quick decline^5^, the oak bacterial leaf scorch^6^, the phony peach disease^7^, the *Citrus* variegated chlorosis CVC^8^ and the almond leaf scorch^9^. As *Xf* induces diseases to a large number of economically important plants including vine^1^, the biology of this pathogen and the mechanisms of vector transmission have been extensively studied to design management strategies^10^.

On the basis of genetic data obtained with Multilocus Sequence Typing MLST^11,12^, *Xf* was subdivided into six subspecies (*fastidiosa*, *morus, multiplex*, *pauca*, *sandyi* and *tashke*). Those subspecies were further characterized by different geographic origins, distributions and host preferences in the Americas^13–15^. However, the intraspecific taxonomic boundaries of *Xf* are still debated^16^ and only the two subspecies *fastidiosa* and *multiplex* are formally considered valid names^1,17^. *Xf* subsp. *fastidiosa*^18^ occurs in North and Central America, where it causes, among others, the harmful PD and the almond leaf scorch (ALS). Genetic analyses suggest that this subspecies originates from southern parts of Central America^19^. The subspecies *multiplex* is widely distributed in North America (from California to western Canada and from Florida to eastern Canada), where it was detected on a wide range of host plants (*e.g*., oak, elm, maple, almond, sycamore, *Prunus* sp., etc.) as well as in South America^20,21^. The subspecies *pauca*, which causes severe diseases in *Citrus* (CVC) and coffee (Coffee Leaf Scorch)^22^ in South and Central America, is speculated to be native to South America^23^. The subspecies *morus* recently proposed by Nunney *et al*.^24^, occurs in California and eastern USA, where it is associated to mulberry leaf scorch. *Xf* subsp. *sandyi*, responsible for oleander leaf scorch, is distributed in California^12^, while the subspecies *tashke* was proposed by Randall *et al*.^25^ for a strain occurring on *Chitalpa tashkentensis* in New Mexico and Arizona. Overall, intraspecific entities of *Xf* display noticeable differences in host range suggesting that the radiation of *Xf* into multiple subspecies and strains is primarily associated to host specialization^26^.

*Xylella fastidiosa* is now of worldwide concern. In 2013, the CoDIRO strain (subsp. *pauca*) was detected on olive trees in southern part of the Apulia territory (Italy). Genetic analyses suggest that this strain was accidentally introduced in Italy from Costa Rica or Honduras via infected ornamental coffee plants^5^. Since then, *Xf* subsp. *pauca* has spread northward and killed millions of olive trees in the Apulia territory, causing unprecedented socio-economic issues. During the period 2015–2017 several subspecies and strains were detected on *ca*. 30 different host plants in Southern France (PACA region) and Corsica^27^. According to national surveys performed in France, the vast majority of plant samples were contaminated by two strains of *Xf* subsp. *multiplex*, and two strains were identified (hereafter referred to as the French ST6 and ST7 strains)^27^. These strains are closely related to the Californian strains Dixon (ST6) and Griffin (ST7), belonging to the “almond group”^26^ and that were detected on numerous plant species, though without evident specialization. To a lesser extent, other strains occur in Southern France *i.e*., the strain ST53 (*Xf* subsp. *pauca)* was detected on *Polygala myrtifolia* in Côte d’Azur (Menton) and on *Quercus ilex* in Corsica^27^ and the recombinants strains (ST76, ST79 or not yet fully characterized) were detected in a few plant samples. In 2016, *Xf* subsp. *fastidiosa* was detected on rosemary and oleander plants overwintering in a nursery in Germany^28^. In 2017, Spanish plant biosecurity agencies officially confirmed the detection of *Xf* strains belonging to the subspecies *multiplex*, *pauca* and *fastidiosa* on almond trees, grapevine, cherry and plums in western parts of the Iberian Peninsula and Balearic islands^29^. Outside Europe, the detection of *Xf* was officially confirmed in Iran on almond trees and grapevines^30^, in Turkey^31^ as well as in Taiwan on grapevines^32^.

The severity of *Xf*-induced diseases has recently increased possibly due to global warming^33^. Indeed, it has been demonstrated that cold winter temperatures might affect the survival of *Xf* in xylem vessels and allow plants to partly recover from *Xf*-induced diseases (‘cold curing phenomenon’)^34,35^. For instance, Purcell^34^ showed that grapevines with symptoms of PD recovered after multiple exposures to temperatures below −8 °C during several hours. Further, Anas *et al*.^36^ suggested that areas experiencing more than 2 to 3 days with minimal temperature below −12.2 °C (or alternatively 4 to 5 days below −9.9 °C) should be considered at low risk for PD incidence, although these thresholds were considered too conservative by Lieth *et al*.^37^. Several studies aimed to forecast the potential distribution of *Xf* in Europe^38^ and/or all over the world^39^. For instance, Hoddle *et al*.^39^ used the CLIMEX algorithm to forecast the worldwide potential severity of PD. Their model suggested that most of Mediterranean areas are suitable for PD even though cold in winter would presumably hamper Xf range expansion into several of the most economically-important wine-producing regions of France and central and northern parts of Spain and Italy. Bosso *et al*.^38^ fitted a Maxent model to forecast the potential distribution of *Xf* subsp. *pauca* under current and future climate conditions, and concluded that climate change would not affect the future distribution of *Xf*. Here, we analyze the potential distribution of three subspecies of *Xylella fastidiosa* using datasets describing native and newly established ranges and a set of four different species distribution models.

## Material and Methods

### Distribution data

**Figure 1:**
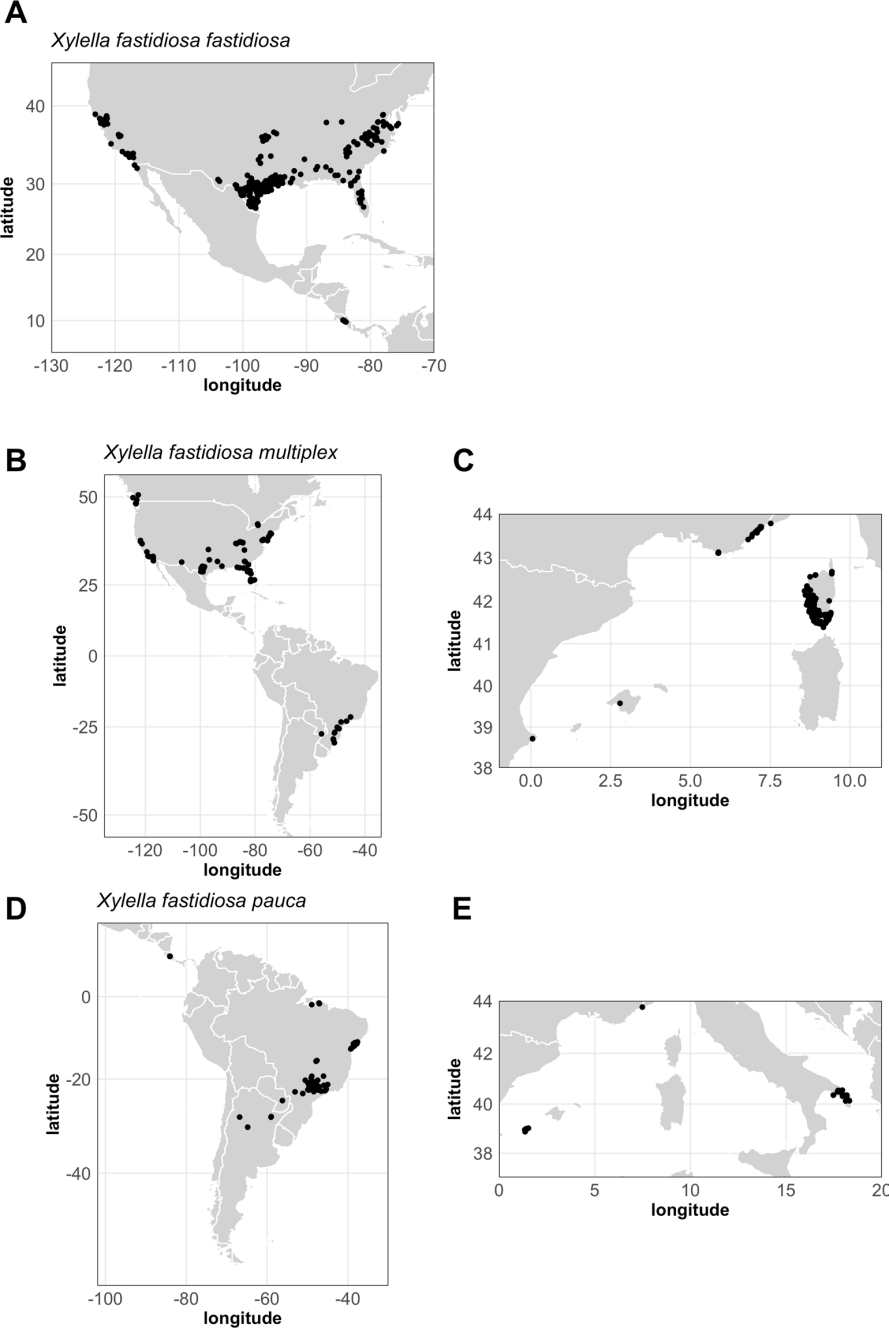
Occurrences of three *Xylella fastidiosa* subspecies used in the study. (**A**) *Xylella fastidiosa fastidiosa*, (**B**) *Xylella fastidiosa multiplex* in its native range and (**C**) in Europe, (**D**) *Xylella fastidiosa pauca* in its native range and (**E**) in Europe. European occurrences of *Xylella fastidiosa fastidiosa* are sparse and not shown.

We collected occurrence data for subspecies *fastidiosa*, *multiplex* and *pauca* from the scientific literature, field surveys and public databases (Fig. 1). These datasets comprised both occurrences from native area (the Americas) and recently invaded regions (south Italy, France and Spain - Fig. 1). The occurrences located in France were collected in 2015–2019 and stored in the French national database managed by the French Agency for Food, Environmental and Occupational Health & Safety (ANSES) (Fig. 1C). For each subspecies, we randomly generated 10,000 background points within a wide area to properly depict the background environment in the Maxent calibration (see below)^40,41^ (Supplementary Fig. S1). Pseudo-absences were randomly generated within regions located in the native area where we could confidently consider that the studied subspecies is absent. We restricted these areas to cold regions because low winter temperatures constitute a well-known factor constraining the distribution of *Xf* ^35^ (Supplementary Fig. S1).

### Bioclimatic descriptors

We used a set of bioclimatic descriptors hosted in the Worldclim database^42^. We retained raster layers of 2.5-minute spatial resolution, which corresponds to about 4.5 km at the equator. The data represent the average climate conditions for the period 1970–2000.

### Models

The potential distribution of *Xf* subsp. *fastidiosa*, *pauca* and *multiplex* were assessed using species distribution modeling. Such approach establishes mathematical species-environment relationships using occurrence/absence records and environmental descriptors in order to assess the potential distribution of species^43^. Different modeling techniques exist and their ability to predict species distribution / habitat suitability is known to vary substantially according to the algorithm used, the occurrence dataset, the environmental descriptors and the model calibration parameters^44,45^. As a consequence, there is no single ‘best’ modeling technique^46^. Such variation has led to the development of ensemble forecasting approach, which aims at building more robust forecasts by combining individual models in a consensus model^47^. However, averaging models’ outputs in the form of probabilities might raise issues. Indeed, models’ response to species prevalence could differ and yield non-comparable probabilities. A solution consists to use a committee averaging approach^44,48^.

In the present study, we adopted a two-step modeling strategy^46^. In the first step of the analysis we tested a set of four algorithms known for their good performance in species distribution modeling (Table 1). The test consisted in (i) fitting models using the occurrences available in the native areas only and (ii) evaluating the predictive power of those models into the recently colonized areas in Europe. It is known that bioclimatic descriptors used to calibrate the models can strongly impact performance and transferability. However, choosing proper descriptors is not easy. Consequently, we constituted seven subsets of bioclimatic descriptors by associating different variables that we *a priori* considered as ecologically meaningful when working on *Xf* (Table 1). We intentionally used a limited number of climate descriptors comprised between two to four to avoid model over-parameterization, which is a recommended practice, particularly when assessing invasion risk^49^. We used two descriptors of the low temperatures during the coldest periods of the year (bio6: minimum temperature of the coldest month; bio11: mean temperature of the coldest quarter). We also used a variable reflecting high temperatures during the warmest period of the year (bio10) and a variable reflecting the rainfall seasonality (bio15) that recently proved to be a good predictor of the spatial distribution of *Xf* in Corsica^50^. For each subspecies of *Xf*, algorithms were calibrated using each climate data subsets and the occurrences available in the Americas. The predictive power of each model was evaluated using the area under the curve of the receiver operating curve (AUC) statistic and the true skill statistic (TSS)^51,52^. This procedure aimed to identify the combinations of algorithms × climate datasets with weak predictive performances and to discard them from further analysis^46^.

**Table 1:** Range of the evaluation metrics for species distribution models calibrated with different climate datasets for *Xylella fastidiosa fastidiosa*, *X. fastidiosa multiplex* and *X. fastidiosa pauca*.

Algorithm	metric	Dataset 1	Dataset 2	Dataset 3	Dataset 4	Dataset 5	Dataset 6	Dataset 7
		bio6	bio6	bio6	bio6	bio6		
		bio10	bio10	bio10	bio10		bio10	bio10
			bio11	bio11			bio11	bio11
				bio15	bio15	bio15		bio15
**Xf fastidiosa**								
Ann	AUC	0.93–0.96	0.94–0.99	0.98–1	0.98–0.99	0.98–0.98	0.89–0.94	0.98–0.99
—	TSS	0.78–0.85	0.81–0.92	0.91–0.95	0.86–0.93	0.78–0.9	0.63–0.82	0.86–0.91
bioclim	AUC	0.92–0.94	0.92–0.94	0.93–0.94	0.93–0.94	0.89–0.93	0.89–0.91	0.89–0.92
—	TSS	0.77–0.86	0.78–0.86	0.79–0.88	0.78–0.88	0.75–0.85	0.71–0.81	0.73–0.82
GLM	AUC	0.92–0.96	0.97–0.97	0.96–0.98	1–1	0.97–0.98	0.93–0.93	0.99–1
—	TSS	0.8–0.9	0.8–0.82	0.75–0.92	0.93–0.97	0.72–0.86	0.61–0.73	0.87–0.91
maxent	AUC	0.94–0.95	0.94–0.94	0.98–0.98	0.98–0.99	0.97–0.98	0.92–0.93	0.94–0.96
—	TSS	0.67–0.82	0.6–0.68	0.79–0.86	0.76–0.9	0.84–0.89	0.66–0.79	0.62–0.69
**Xf multiplex**								
Ann	AUC	0.99–1	0.99–1	1–1	1–1	1–1	1–1	0.99–1
—	TSS	0.94–0.97	0.96–0.99	0.95–1	0.99–1	1–1	1–1	0.94–1
bioclim	AUC	0.94–1	0.94–1	0.9–0.99	0.9–0.99	0.94–0.99	0.93–0.99	0.88–0.98
—	TSS	0.82–0.95	0.79–0.93	0.7–0.9	0.73–0.92	0.79–0.94	0.76–0.89	0.65–0.84
GLM	AUC	1–1	0.97–0.98	0.93–0.98	0.97–0.98	1–1	1–1	1–1
—	TSS	0.89–0.97	0.92–0.93	0.86–0.93	0.93–0.97	0.98–1	0.94–1	0.97–1
maxent	AUC	0.99–1	0.88–0.92	0.9–0.93	0.86–0.89	0.99–1	0.98–0.99	0.9–0.94
—	TSS	0.73–0.89	0.64–0.64	0.6–0.74	0.62–0.74	0.77–0.89	0.79–0.86	0.7–0.87
**Xf pauca**								
Ann	AUC	1–1	1–1	0.97–0.97	1–1	1–1	NC	0.97–1
—	TSS	0.99–1	0.99–1	0.94–0.94	1–1	0.99–1	NC	0.94–0.94
bioclim	AUC	0.92–1	NC	NC	0.92–1	1–1	NC	NC
—	TSS	0.85–1	NC	NC	0.85–1	0.98–1	NC	NC
GLM	AUC	1–1	NC	NC	0.97–1	1–1	NC	NC
—	TSS	0.99–1	NC	NC	0.94–1	1–1	NC	NC
maxent	AUC	0.98–0.99	0.88–0.93	1–1	0.99–0.99	0.99–0.99	0.99–1	0.99–1
—	TSS	0.69–0.94	0.68–0.8	0.77–0.98	0.78–0.92	0.77–0.9	0.86–0.9	0.88–0.97

For each algorithm and each dataset five models based on a subset of 80% randomly selected presence data were calibrated. We report the range of the metrics for models retained in the computation of the consensus models (TSS >0.6 and auc >0.85). bio6: minimum temperature of the coldest month; bio10: mean temperature of warmest quarter; bio11: mean temperature of the coldest quarter; bio15: precipitation seasonality. NC: not computed.

In a second step of the analysis we used the best combinations of algorithms × climate datasets to calibrate models with all available subspecies occurrences to predict habitat suitability in Europe (referred to as full models). For each combination of model and climate datasets we calibrated the models using a randomly selected subset of eighty percent of the occurrence data (native and invaded ranges) and used the remaining twenty percent for model evaluation using AUC and TSS metrics. Five replications were done for each full model. Evaluations were done using a set of 100 randomly generated pseudo-absences.

We removed autocorrelated data to improve model predictive performances^53,54^. To do so, we used the first two axes of a Principal Component Analysis performed on the bioclimatic variables^55^ recorded at each occurrence points. The first axis was divided into 100 bins. The bins of the second axis were fixed to have the same amplitude as for axis 1. The occurrence points were projected onto the resulting grid. When a grid cell contained more than one point, a random selection was used to retain only one point for further model calibration. This procedure was repeated for each *Xf* subspecies and for each climate dataset.

Model outputs were transformed into binary projections using the threshold that optimized the TSS statistics on the testing data^56^. The resulting projections were averaged to compute the committee (consensus) averaging that shows the likelihood of the presence of a species given the available data. The consensus model ranges from 0 (all the models predict absence) to 100 (all the models predict presence)^44,56^. We removed the individual models that did not reach the minimum quality threshold of TSS >0.6 and AUC >0.85^56^ before computing the consensus (Table 1).

Our set of four algorithms comprised approaches belonging to the three main functional groups of species distribution models^57^. First, we selected the envelope model Bioclim^58,59^ that relies only on presence data and as such makes no assumption about the absence of the organism under study^60^. Second we employed the maximum entropy algorithm Maxent that discriminates presences with background data^61^. Third, we used modeling techniques that rely on presence and absence or pseudo-absences namely the generalized linear model (GLM)^62^ and the artificial neural network^56,63^. Maxent was fitted using 10,000 background points while GLM and artificial neural network were fitted using 200 pseudo-absences (Fig. S1).

Caution is usually warranted when interpreting models projected into new areas with climate conditions different from the calibration area^64–66^. Thus, we assessed the similarity of climate conditions between the calibration dataset and the projected area (*i.e*., Europe) by computing the MESS index^41^.

The following R^67^ packages were used to perform analyses and generate graphical outputs: biomod2^68^, cowplot^69^, dismo^70^, ecospat^71^, ggplot2^72^, raster^73^ and rmaxent^74^.

## Results

### Algorithm and climate datasets selection

The best combinations of algorithms × climate datasets for *Xf multiplex* and *Xf pauca* were evaluated with a dataset comprising all European occurrences. As there were too few occurrences in Europe for *Xf fastidiosa*, a random subset of 20% of the native range occurrences was used. Models were selected on the basis of an arbitrary threshold of the TSS fixed to 0.6^56^. Only the following combination did not get sufficient statistical support to be retained in future analysis of the potential distribution of *Xf pauca*: bioclim × dataset 2, 3, 6 and 7; GLM × dataset 2, 3, 6 and 7 and Ann × dataset 6 (Table 1). The remaining combinations were used to calibrate the full models based on the complete set of occurrences.

**Figure 2:**
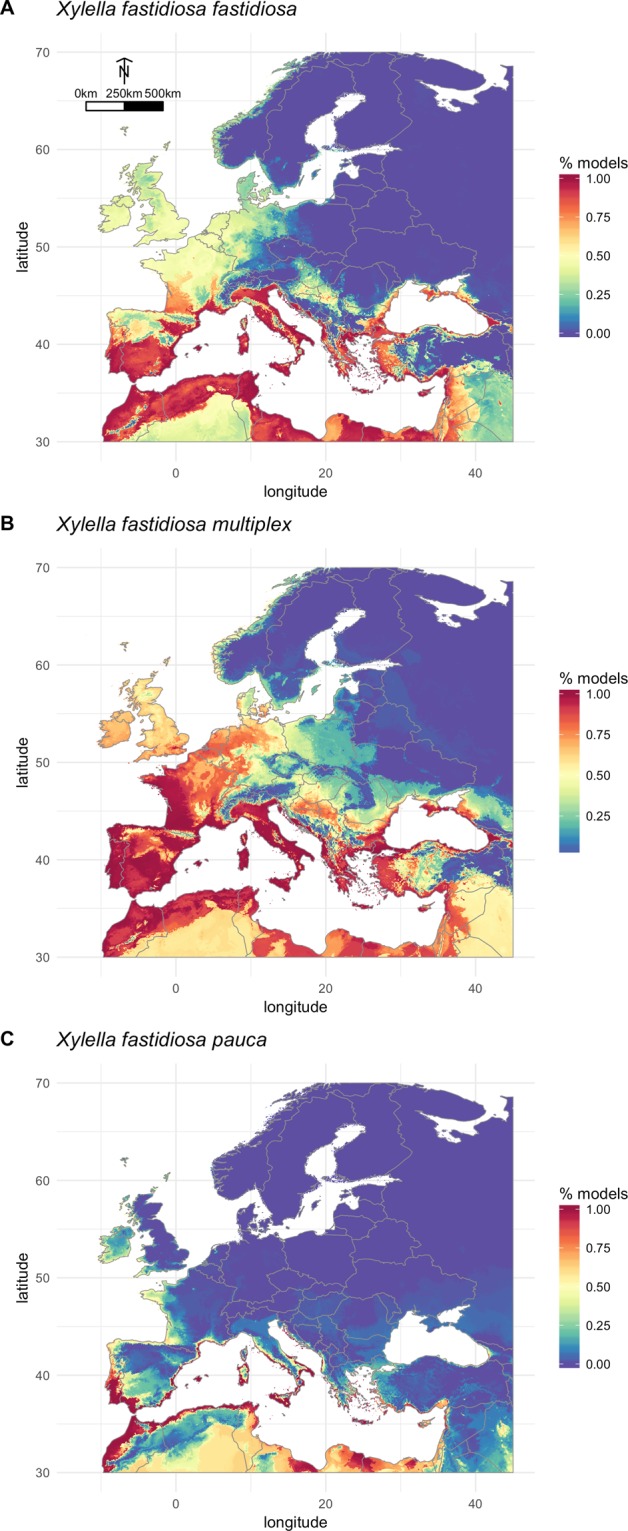
Potential distribution of three subspecies of *Xylella fastidiosa*: (**A**) Xf subspecies *fastidiosa* (**B**) Xf subspecies *multiplex* (**C**) Xf subspecies *pauca*. Maps depict the ensemble forecast derived from committee averaging based on lowest presence thresholding (see methods section for details). The index varies from 100 when all models predict presence to 0 if all the models predict absence of the subspecies.

The accuracy of the resulting full models was examined and we only retained the full models associated to TSS >0.6 and AUC >0.85 for the computation of the consensus model. This quality check resulted in discarding seven, four and four models for *Xf fastidiosa, Xf multiplex* and *Xf pauca* respectively. Table 1 shows the range of the evaluation metrics for the models used to compute the consensus models (Fig. 2).

### Potential distribution of Xylella fastidiosa

Figure 2 shows the committee averaging for the three subspecies of *Xf*. Each map shows the proportion of models predicting the presence of *Xf* in Western Europe. The potential distribution of *Xf* subsp. *fastidiosa* includes large regions of Spain, France, Italy, Croatia, Greece and Turkey as well as the coastal regions of North Africa (Fig. 2A). The proportion of models indicating favorable climate conditions in these areas was close or equal to 100%. The agreement between algorithms × climate datasets was somewhat lower in the northern part of France, the British Islands, Belgium and Netherlands where a lower proportion of models predicted the presence of *Xf* subsp. *fastidiosa*. In the case of *Xf* subsp. *fastidiosa* (Fig. 2A), the MESS index indicated that climate conditions encountered within Western Europe do not differ from the climate conditions that characterize the native area (Supplementary Fig. S2).

The predicted potential distribution of *Xf* subsp. *multiplex* is depicted in Fig. 2B. European climate appears favorable in a very large area covering Spain, France, the British Isles, Italy, the Adriatic coast, Greece, Turkey and some coastal areas of the Black Sea. North Africa and Mediterranean coast of near East countries are also climatically suitable. The MESS index indicated a good match between the range of climate conditions that prevail in Europe and in the American range of *Xf* subsp. *multiplex* (Supplementary Fig. S3).

The predicted extent of climatically suitable conditions for *Xf* subsp. *pauca* is limited to the Mediterranean coastal regions with the exception of south Portugal and Spain Atlantic coasts. The MESS index indicated a mismatch between the minimum temperatures (bio6 and bio11) in northern Europe and the native area (Supplementary Fig. S4).

## Discussion

### Geographical distribution and possible impacts in Europe

In a rapidly changing world, the design of pest control strategies (*e.g*., early detection surveys and planning of phytosanitary measures) should ideally rely on accurate estimates of the potential distribution and/or impact of pest species as well as their responses to climate change^75^. In the present study, bioclimatic models predicted that a large part of the Mediterranean lowlands and Atlantic coastal areas of Europe are characterized by climatically suitable conditions for *Xf* subsp. *fastidiosa, multiplex* and *pauca* (Fig. 2). To a lower extent, favorable climate suitability is also observed in northern and eastern regions of Europe (North-eastern France, Belgium, the Netherlands, Germany, etc.).

Our models displayed good evaluation measures and predicted high climatic suitability in all European areas where symptomatic plants are currently infected by the subspecies *fastidiosa, multiplex* or *pauca* (*e.g*., Balearic Islands, lowlands of Corsica island, south-eastern France and the Apulia region). This suggests that climate suitability maps provided in the present study are reliable for the design of further sampling strategies, including ‘sentinel insects’ survey^76,77^. They may also be helpful to anticipate the spread of the different subspecies and provide guidance on which areas should be targeted for an analysis of local communities of potential vectors and host plants and to design further management strategies and research projects.

The results show that the different subspecies of *Xf* studied here might significantly expand in the near future, irrespective of climate change. For example, the subsp. *multiplex* known from Corsica and southern France have a large potential for expansion in Europe (Fig. 2B), which is not surprising since this subspecies has a wide distribution, ranging from Florida to Canada^78^. Actually, its expansion probably depends more on plant exchanges, vector spatio-temporal patterns and disease management than on climate suitability *per se*. *Xf multiplex* is associated to economically important plants such as almonds and olives^26^ but may also colonize multiple ornamental plants or forest species^27^. Its potential distribution in Europe extends far beyond areas where the subspecies has been reported which suggests that new outbreaks may occur that could result in important economic losses.

The subspecies *fastidiosa*, which has been currently reported from a limited number of localities in Europe, could encounter favorable climate conditions in various areas (Fig. 2A). Notably, the models predict climate suitability in strategic wine-growing areas in different countries. The herein estimates of the potential distribution of the *subsp. fastidiosa* are consistent with the risk maps provided by Hoddle *et al*.^39^ and Purcell (available in Anas *et al*.^36^).

The case of subsp. *pauca* is somewhat different (Fig. 2C). Most of the European occurrences come from southern Italy and the Balearic Islands and the potential distribution of this subspecies appears limited. Nevertheless, southern Spain and France, Portugal, Corsica, Sardinia, Sicilia and North Africa that are areas where growing olive trees is multisecular offer suitable conditions, which potentially implies huge socio-economic impacts.

One factor that proved to be critical for some insect-borne plant diseases is the distribution/availability of vectors and hosts. Here, none of these factors is limiting in most of Europe since *Xf* is capable of colonizing a vast array of plants present in Europe and *Philaenus spumarius*, the putatively most efficient European vector so far^79,80^, occurs across most of the continent^77^.

The potential distribution of the three subspecies of *Xf* studied here appeared to be limited by minimum winter temperatures with *Xf* subsp. *pauca* being much more sensible than the others. Because “cold curing” appears to be the main regulating mechanism, is it very likely that climate change would alter the distribution of suitable areas for *Xf* in Europe as the minimum winter temperatures might increase^34,35,81^. Furthermore, the potential dynamics of *Xf* in areas experiencing extremely high temperatures in summer (*e.g*., southern and central Spain) remain largely uncertain as the impact of extreme heat on *Xf* is poorly known^82^. Although warm spring and summer temperatures enhance multiplication of *Xf* in plants, it has been showed that *Xf* populations decrease in grapevines exposed to temperatures above 37 °C^35^. As southern and central Spain frequently experience temperatures above 40 °C in summer, additional data would be helpful to better understand the potential distribution and impact of *Xf* in these regions. Another point requiring clarification is the effect of rain and moisture. As Xf bacteria live in the xylem of plants they are subject to stress whenever their host is itself under water-stress. Precipitation or moisture may also have indirect impacts on Xf through insect vectors whose activity or behavior could be altered by water stress^83^. Although the three subspecies of *Xf* seem to have different tolerances to cold, it is, however, unclear whether realized niche divergence among subspecies reflects inherent differences in thermal tolerances or rather host-pathogen interactions as it was observed for *Ralstonia solanacearum*^84^. Additional investigations would allow a better understanding of the effect of temperatures on the different strains of *Xf*. It is noteworthy that potential distributions show large areas of potential co-occurrence. This may have important implications as it may increase the risk of intersubspecific homologous recombination^11^.

### Limits and opportunities for risk assessment

Maps of habitat suitability should be cautiously interpreted as they are derived from correlative tools that depict the *realized* niche of species *i.e*., a subset of the *fundamental* environmental tolerances constrained by biotic interactions, landscape structure and dispersal limits^85^. In addition, time-periods associated to occurrences and climate descriptors dataset do not perfectly overlap. The models were fitted with climate descriptors that represent average climate conditions for the 1970–2000 period, while some presence records were collected after 2000 in a period characterized by milder winter temperatures. Moreover, we deliberately fitted the models using a few climate descriptors to avoid model over-parameterization and/or extrapolation and enhance model transferability. Consequently, we cannot exclude that bioclimatic models presented here did not fully capture the entire range of environmental tolerances and did not fully depict the complexity of the climatic niche of *Xf* as well as potential interactions between climate descriptors. Better models and hence, better risk assessment could be obtained by collecting additional occurrence data as well as reliable absence data. The possible adaptation of *Xf* to new environmental constraints in its invaded range (*e.g*., by genetic recombination) is another important source of uncertainty. Finally, it is worth noting that bioclimatic models predict climatic suitability of a geographic region for *Xf* rather than a proper risk of *Xf*-induced disease incidence. To predict the proper severity of *Xf*-induced diseases in a given locality, statistical models should account for many additional factors playing a role in *Xf* epidemiology, including *e.g*., microclimate conditions, inter-annual climate variability, host-plant sensitivity, host-pathogen interactions, landscape structure and the spatio-temporal structure of the community of potential vectors. Although recent entomological studies identified the meadow spittlebug *P. spumarius* as the main vector of *Xf* in Italy^79,80^, a better knowledge of all European vectors capable of transmitting *Xf* to plants as well as their ecological characteristics (geographic range, efficiency in *Xf* transmission, demography, overwintering stage, intra-specific diversity, etc.) is needed^86^. In this context, habitat suitability maps could allow to design cost-efficient vector surveys, with priority given to geographic regions predicted as highly climatically suitable for *Xf*. The study by Cruaud *et al*.^77^ provides a good insight into how species distribution modeling and DNA sequencing approaches may be combined for an accurate monitoring of the range of *Xf* and its vectors in Europe. We believe that SDMs are valuable tools to help in designing research experiments, control strategies as well as political decisions at the European scale.

## Conclusions/highlights

Species distribution models all indicate that the currently reported geographical range of *Xf* in Europe is small compared to the large extent of climatically suitable areas. This is true for all studied subspecies of *Xf* although the subspecies *pauca* appears to have a smaller potential range. *Xf* has a certain potential to adapt to climate and biotic conditions (hosts, vectors) encountered in Europe. The magnitude of this adaptive potential remains largely unknown but could nevertheless lead to a substantial spread of this plant pathogen across Europe. A further important research effort is thus needed to decipher the potential host plants – insect vectors – bacterium interactions in the (sub)natural ecosystems as well as agro-ecosystems at risk^55^. Only in this way could we develop an appropriate and efficient strategy to control *Xf* in the future.

## Supplementary information

supplementary information

## Data Availability

The occurrence data in France were made available to the authors by the French Ministère de l′Agriculture et de l′Alimentation subject to confidentiality requirements. The occurrence data in North and South America are available upon request to the authors.
