# Supplementary material for: Xylella fastidiosa: climate suitability of European continent
Source: Sci Rep. 2019 Jun 20;9:8844. doi: 10.1038/s41598-019-45365-y (PMC6586794; doi:10.1038/s41598-019-45365-y)

Supplementary data to

***Xylella fastidiosa*: climate suitability of European continent**

Martin Godefroid, Astrid Cruaud, Jean-Claude Streito, Jean-Yves Rasplus and Jean-Pierre Rossi

Figure S1: Maps of background data and pseudo-absences for 3 subspecies of *Xylella fastidiosa.* A. *Xylella fastidiosa fastidosa* B. *Xylella fastidiosa multiplex* C. *Xylella fastidiosa pauca*

The MESS index

Figure S2: Maps of the multivariate environmental similarity surface (MESS) computed for the occurrence points of *Xylella fastidiosa fastidiosa.* Values < 0 indicate areas where the model predictions were computed for environmental conditions absent from the occurrence dataset.

Figure S3: Maps of the multivariate environmental similarity surface (MESS) computed for the occurrence points of *Xylella fastidiosa multiplex.* Values < 0 indicate areas where the model predictions were computed for environmental conditions absent from the occurrence dataset.

Figure S4: Maps of the multivariate environmental similarity surface (MESS) computed for the occurrence points of *Xylella fastidiosa pauca.* Values < 0 indicate areas where the model predictions were computed for environmental conditions absent from the occurrence dataset.

Figure S1

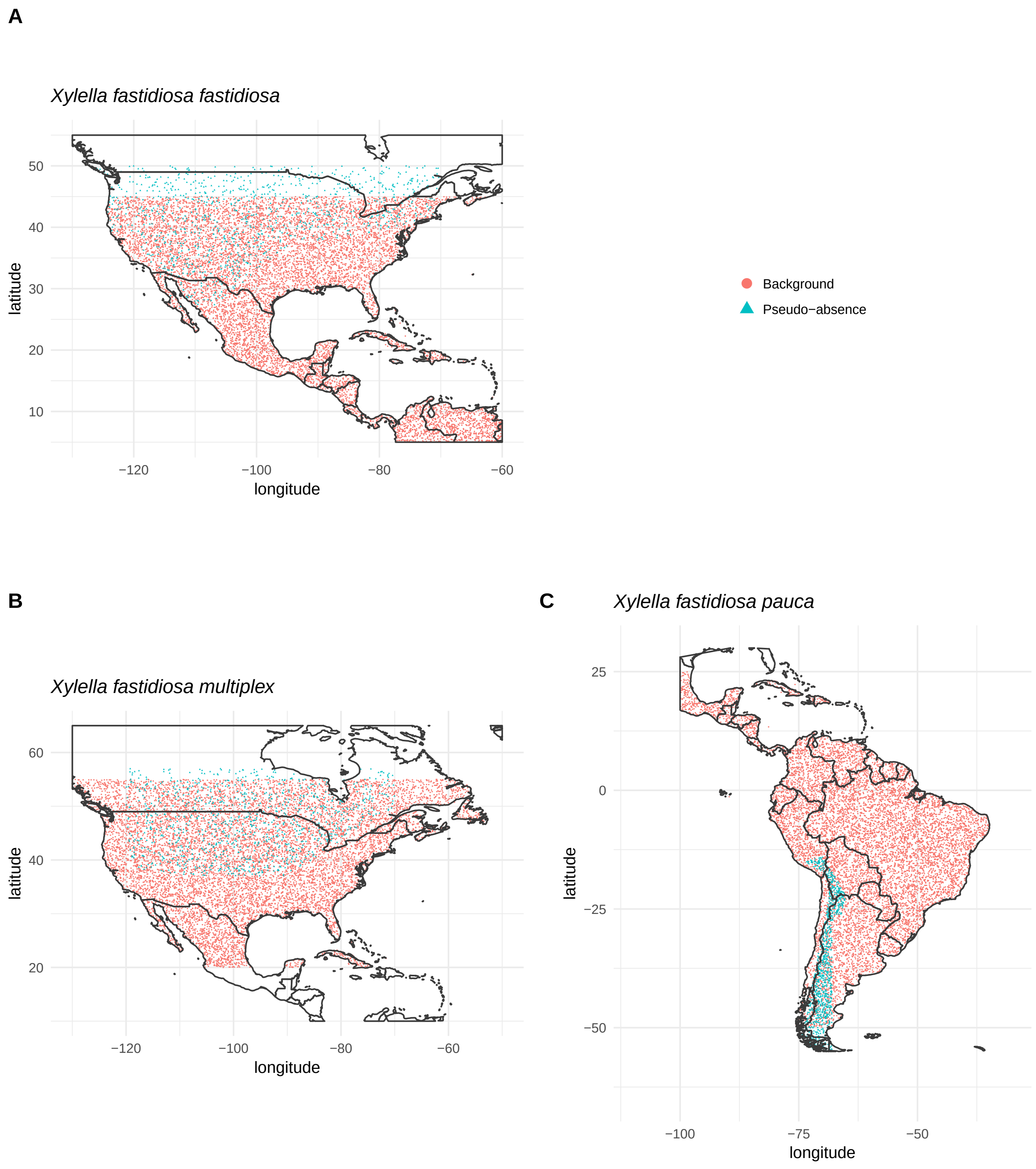
A
Xylella fastidiosa fastidiosa
latitude
longitude
Background
Pseudo−absence
B
Xylella fastidiosa multiplex
latitude
longitude
C
Xylella fastidiosa pauca
latitude
longitude

The MESS index

MESS stands for Multivariate Environmental Similarity Surfaces (Elith et al 2010). It is an index of similarity reporting the closeness of a point described by a set of variables to the distribution of these variables within a population of reference points. The index is computed for each variable (Figure S2 B-E) and the multivariate similarity (Figure S2 A) is the minimum value of the similarity with respect to each variable. The lower the index associated to a pixel, the larger the difference between the environment at the pixel and the environment characterizing the set of reference points. Negative values indicate that at least one variable has a value situated outside the range of the reference set. This allows identifying areas with environmental conditions different from the dataset used to calibrate the models. In our study, the reference sets of points are the sets of localities where sub-species of *Xylella fastidiosa* have been detected and a random set of 500 background points. We computed the index for each pixel where an estimation of habitat suitability has been computed using the species distribution models.

Reference:

Elith, J., Kearney, M. & Phillips, S. 2010. The art of modelling range-shifting species. Methods Ecol. Evol. 1, 330-342.

Figure S2. Maps of the multivariate environmental similarity surface (MESS) computed for the occurrence points of *Xylella fastidiosa fastidiosa*

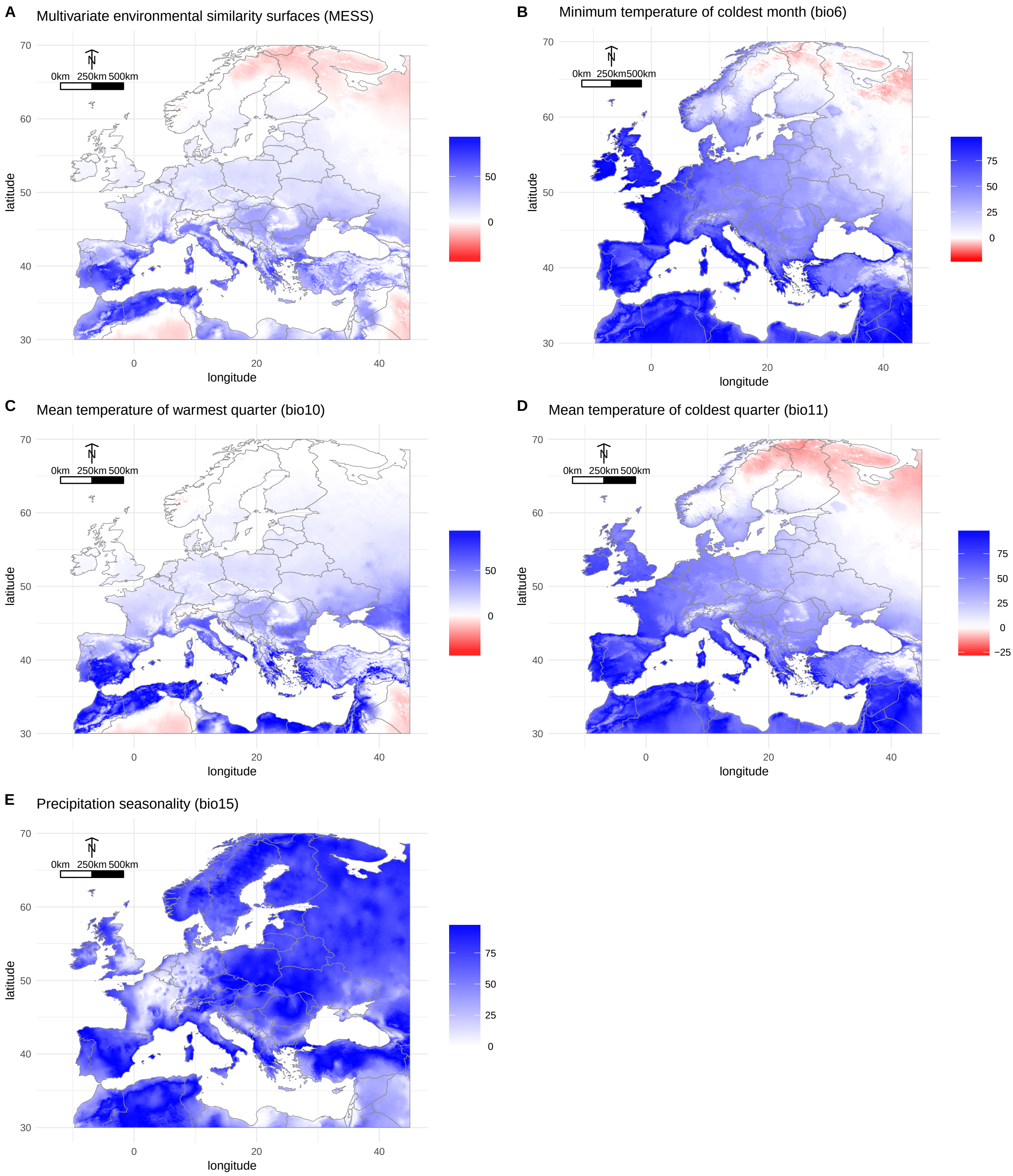

Figure S3. Maps of the multivariate environmental similarity surface (MESS) computed for the occurrence points of *Xylella fastidiosa multiplex*

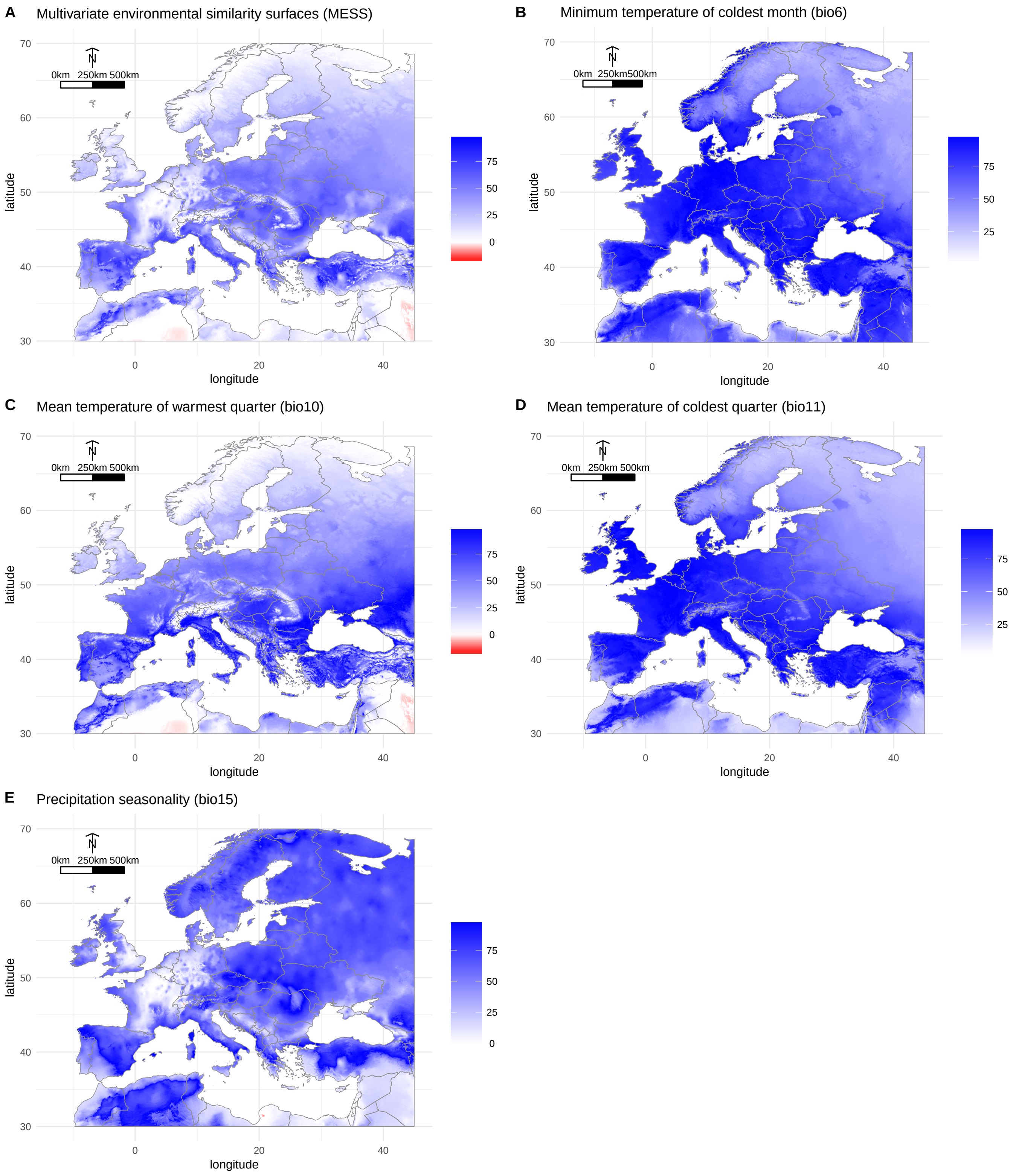

Figure S4. Maps of the multivariate environmental similarity surface (MESS) computed for the occurrence points of *Xylella fastidiosa pauca*

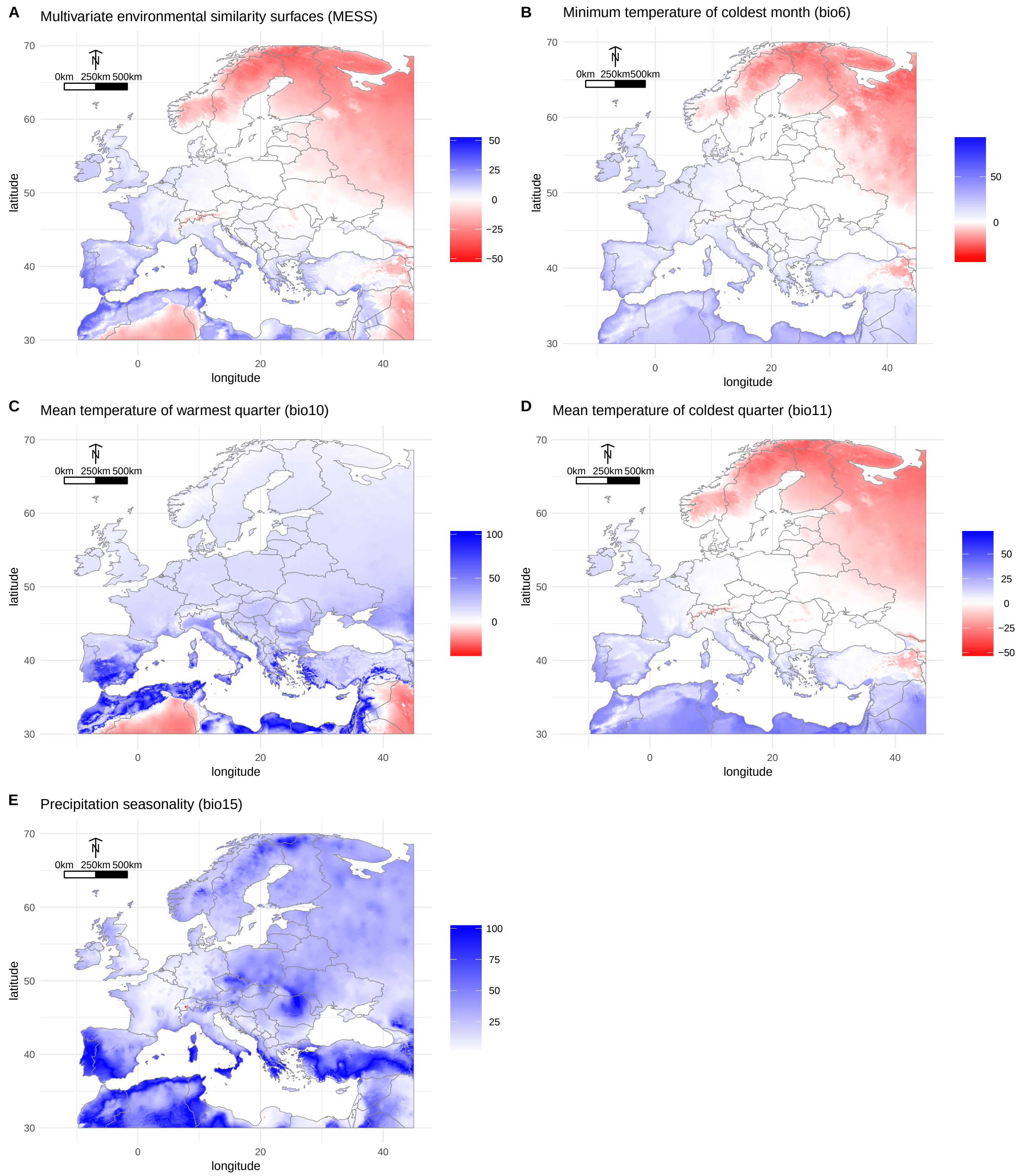

Supplement: Supplementary file 1 — supplementary information [file 41598_2019_45365_MOESM1_ESM.pdf]
